# Outcomes of Anterior Cruciate Ligament Reconstruction in Patients with Associated Anterolateral Ligament Injury

**DOI:** 10.1055/s-0044-1785516

**Published:** 2024-06-22

**Authors:** João Paulo Fernandes Guerreiro, Larissa Baldow Rosa, Ellen Liceras Gonçalves, Amon Ramysés Rodrigues Curcio, Paulo Roberto Bignardi, Marcus Vinicius Danieli

**Affiliations:** 1 Faculdade de Medicina, Pontifícia Universidade Católica do Paraná (PUCPR) – Câmpus Londrina, Londrina, PR, Brasil; 2 Hospital de Ortopedia Uniort.e, Londrina, PR, Brasil; 3 Departamento de Ortopedia e Traumatologia, Faculdade de Medicina, Pontifícia Universidade Católica do Paraná (PUCPR) – Câmpus Londrina, Londrina, PR, Brasil; 4 Serviço de Ortopedia e Traumatologia, Hospital Evangélico de Londrina, Londrina, PR, Brasil

**Keywords:** anterior cruciate ligament, anterolateral ligament, articular ligaments, knee

## Abstract

**Objective** To evaluate if there is a significant difference in the outcomes of isolated anterior cruciate ligament (ACL) reconstruction in patients with or without associated anterolateral ligament (ALL) injury.

**Methods** We conducted a retrospective cross-sectional study through the analysis of medical records and the application of the questionnaires of the Lysholm Knee Scoring Scale and the International Knee Documentation Committee (IKDC) Subjective Knee Form to patients undergoing isolated ACL reconstruction.

**Results** The 52 participants included were divided into two groups: 19 with associated ALL injury and 33 with no associated ALL injury. None of the patients with associated ALL injury suffered an ACL rerupture, and 21.1% presented injuries to other knee structures after surgery. Among the patients with no associated injury, 6.1% suffered ACL rerupture, and 18.2% presented injuries to other structures after surgery ( *p* = 0.544). Return to activities at the same level as that of the preoperative period occurred in 60% of the patients with associated ALL injury and in 72% of those with no associated injury ( *p* = 0.309). The mean score on the Lysholm Knee Scoring Scale was of 81.6 points in patients with associated ALL injury, and of 90.1 in those with no associated injury ( *p* = 0.032). The mean score on the IKDC Subjective Knee Form was of 70.3 points in patients with associated ALL injury and of 76.7 in those with no associated injury ( *p* = 0.112).

**Conclusion** There was no statistically significant difference regarding graft injuries or new injuries to other structures, satisfaction with the operated knee, or the score on the IKDC Subjective Knee Form. Return to activity was similar in the groups with and without associated ALL injuries. The scores on the Lysholm Knee Scoring Scale were better, with a statistically significant difference in the group with no associated ALL injuries.

## Introduction

Anterior cruciate ligament (ACL) injuries are common, and their incidence increases among the physically-active population, with significant consequences for the quality of life, activity index, joint stability, functionality, and risk of development of osteoarthritis. 1 However, even though surgical reconstruction is the treatment of choice, the success rate is influenced by patient- or graft-specific risk factors. 1 2

In recent years, several studies 3 4 have sought to better characterize the anterolateral complex of the knee to minimize such risks and seek more effective treatment; the anterolateral ligament (ALL) has a critical stabilizing function for internal knee rotation, along with the ACL. Injuries to the ACL and ALL present worse postoperative outcomes compared with those of surgical ACL reconstruction alone due to the need to associate extra-articular procedures with intra-articular reconstruction. 5 6

Based on this information, the present study retrospectively analyzed the outcomes of isolated ACL reconstruction in groups of patients with ACL injury associated or not with ALL injuries.

## Materials and Methods

The present study was conducted after approval by the institutional Ethics in Research Committee (CAAE- 61209722.0.0000.0020) and after the participants signed an informed consent form, and it follows resolution no. 466/2012 of the Brazilian National Health Council and the Declaration of Helsinki.

We performed an analysis of medical records and applied the questionnaires of the Lysholm Knee Scoring Scale and of the International Knee Documentation Committee (IKDC) Subjective Knee Form in their Portuguese versions 7 8 to all patients undergoing isolated ACL reconstruction by the same knee surgery group in 2019.

The study included patients with at least two years of follow-up, who had magnetic resonance imaging (MRI) scans performed in the acute phase of the injury (up to three weeks after the initial sprain) and underwent surgery within the first three months after the injury. Patients who refused to participate in the research or were unable to be contacted were excluded.

In the medical records, we collected data on sex, date of birth, date of injury, date of preoperative MRI scan, date of surgery, and operated side. The selected patients were interviewed and filled out the questionnaires. Initially, the participants answered whether they had a new injury to the ACL or to another structure that required surgery on the same knee. Those who did not have new injuries were asked about their satisfaction level with the surgery (very satisfied, satisfied, somewhat satisfied, or dissatisfied) and return to sports (better than before, the same as before, worse than before, or unable to return). In addition, they answered the Lysholm Scale and IKDC functional questionnaires. 7 8 Patients with new injuries were excluded from this stage because they did not meet the criterion of a minimum two years of postoperative follow-up.

### Statistical Analysis

The analysis of the qualitative variables used the Chi-squared (χ ^2^ ) or the Fisher exact test. For the quantitative variable the Shapiro-Wilk normality test. Then, the Mann-Whitney test for non-normal data and the *t* -test for variables with Gaussian distribution. The results were analyzed though the IBM SPSS Statistics for Windows (IBM Corp., Armonk, NY, United States) software, version 23.0, with a confidence level of 5% for all tests applied.

## Results

**Fig. 1:**
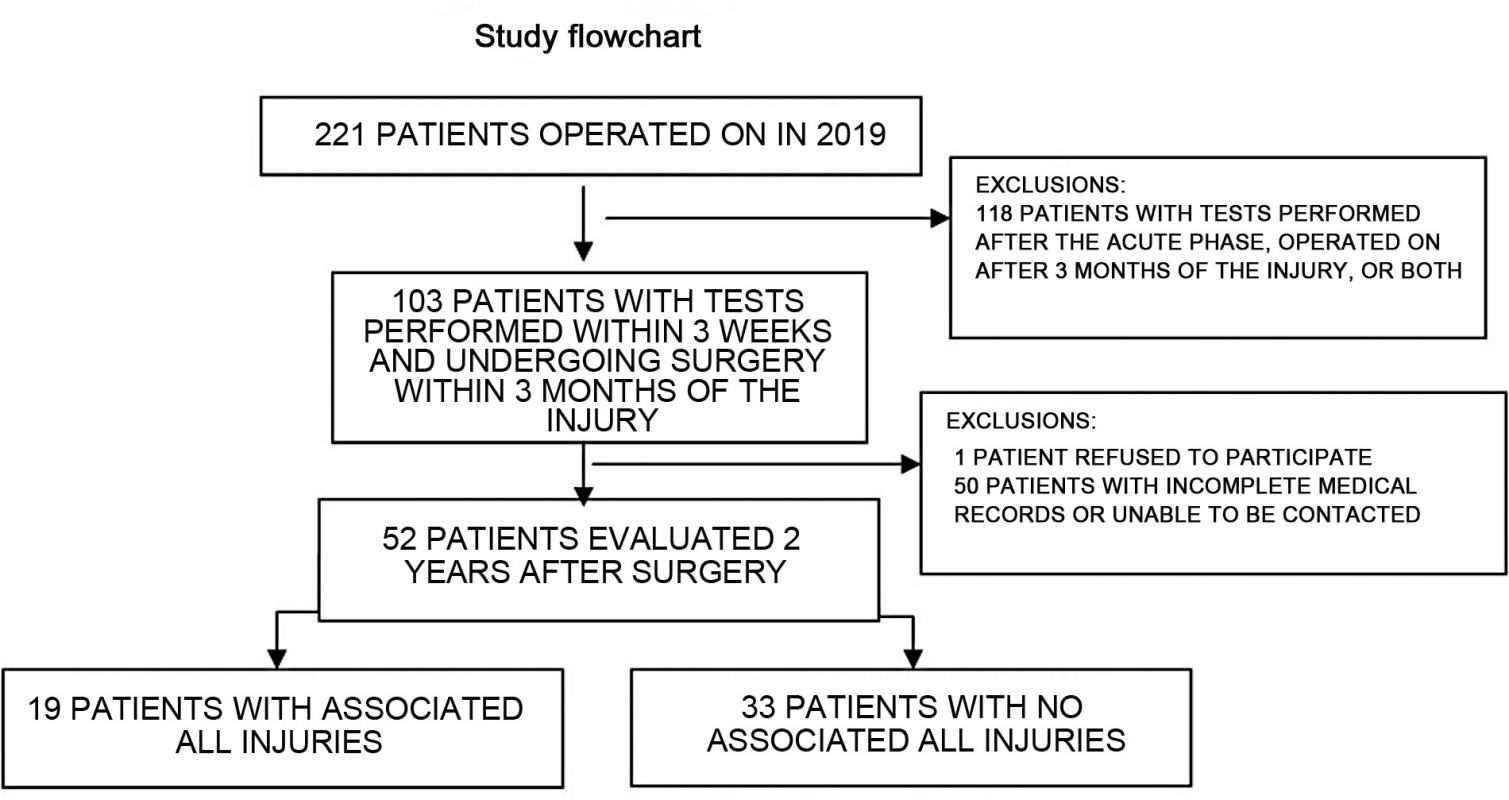
Study flowchart. **Abbreviation:** ALL, anterolateral ligament.

From the initial sample of 221 patients undergoing ACL reconstruction, we included 103 participants with MRI scans performed within the first 3 weeks who underwent surgery within 3 months after the injury. We excluded 1 patient who refused to participate in the research and 50 patients with incomplete medical records and whom we were unable to contact. The 52 participants evaluated were divided into 2 groups according to the presence or absence of ALL injuries before ACL reconstruction surgery ( Fig. 1 ).

The mean age of the patients was of 33.3 years for those with an associated ALL injury and of 38 years for those with no associated ALL injury. The sample was predominantly male, with 73.7% of male patients with an ALL injury and 87.9% of male subjects with no ALL injury. Regarding new injuries after 2 years, none of the patients with an ALL injury presented a new ACL injury, and 21.1% of patients had injured another knee structure (meniscus, cartilage, or another ligament). In patients without an ALL injury, 6.1% had a new ACL injury, and 18.2% injured another structure ( Table 1 ).

**Table 1:** Age, sex, and new injuries

Variable	With ALL injury (n = 19)	Without ALL injury (n = 33)	*p* -value
Age (years): mean ± SD	33.3 ± 8.8	38 ± 10.4	0.243
Male sex: n (%)	14 (73.7)	29 (87.9)	0.260
New knee injury: n (%)			
- Yes, new ACL injury - Yes, in another structure - No new injury	0 (0) 4 (21.1) 15 (78.9)	2 (6.1) 6 (18.2) 25 (75.8)	0.544

Abbreviations: ACL, anterior cruciate ligament; ALL, anterolateral ligament; SD, standard deviation.

The mean age of the patients with no new knee injury was of 34.4 years among those with an associated ALL injury and of 39.6 years in those with no associated injuries. The sample remained predominantly male, with 73.3% of male patients with an ALL injury and 84% of male subjects with no ALL injury. As for satisfaction with the operated knee, most participants from both groups were very satisfied, including 46.7% with an ALL injury and 52% with no ALL injury ( *p* = 0.367).

Regarding return to activities, 60% of the patients with ALL injuries and 72% of those without them ( *p* = 0.309) resumed them at the same level as in the preoperative period.

As for the functional questionnaires, the mean score on the Lysholm Scale was of 81.6 points among patients with an ALL injury, and of 90.1 points in those with no ALL injury ( *p* = 0.032), and the mean IKDC score was of 70.3 points in patients with an ALL injury and if 76 .7 points in subjects with no ALL injury ( *p* = 0.112) ( Table 2 ).

**Table 2:** Satisfaction, return to sports, and functional scores

Variable	With ALL injury (n = 19)	Without ALL injury (n = 33)	*p* -value
Age (years): mean ± SD	34.4 ± 9.4	39.6 ± 10.5	0.118
Male sex: n (%)	11 (73.3)	21 (84)	0.444
Satisfaction: n (%)			
- Very satisfied - Satisfied - Somewhat satisfied - Dissatisfied	7 (46.7) 5 (33.3) 3 (20) 0 (0)	13 (52) 10 (40) 1 (4) 1 (4)	0.367
Return to activities: n (%)			
- Better than before - Same as before - Worse than before - Unable to return	2 (13.3) 9 (60) 4 (26.7) 0 (0)	3 (12) 18 (72) 2 (8) 2 (8)	0.309
Lysholm Knee Scoring Scale: mean ± SD	81.6 ± 18.6	90.1 ± 15.5	0.032
IKDC Subjective Knee Form: mean ± SD	70.3 ± 15.4	76.7 ± 14.3	0.112

Abbreviations: ACL, anterior cruciate ligament; ALL, anterolateral ligament; IKDC, International Knee Documentation Committee; SD, standard deviation.

## Discussion

Analyzing the new ACL graft injury rate, the present study we did not observe significant differences or the need for new surgeries in patients with or without associated ALL injuries. However, some studies 6 9 10 have shown that an associated injury increases the rates of ACL graft rupture and reoperation. However, a large retrospective study 11 with combined reconstruction did not reveal a significant difference concerning isolated ACL reconstructions. The disadvantages of associated injury and advantages of combined reconstruction would result from the anatomical and biomechanical properties of the ALL in terms of rotational stabilization of the knee along with the ACL. Cadaveric studies 12 13 14 have shown that recovery of knee biomechanics and kinetics to preinjury levels only occurs in combined reconstructions of associated injuries.

Some researchers 14 15 16 17 18 consider the presence of a higher criterion for increased risk of ACL rerupture, that is, postoperative positive residual pivot, or two minor criteria for increased risk of reinjury as an indication for combined reconstruction. However, to date, there has been no standardization of the indications for extra-articular ALL reconstruction associated with intra-articular ACL reconstruction, with several authors 3 5 14 15 16 17 18 highlighting the need for more robust studies on the topic.

Regarding functional results and postreconstruction patient satisfaction, most studies 10 14 15 16 17 18 19 20 21 22 23 show objective and/or subjective improvement with combined reconstruction. Nevertheless, there is still much controversy in the literature, with most studies showing no statistically significant difference, except regarding the presence of joint hypermobility associated with significant knee rotational instability, in which combined reconstruction has led to better patient satisfaction. 10 16 17 19 21 As far as the return to daily activities and sports, combined reconstruction has resulted in functional improvement, especially in populations with knee hypermobility. 15 16 17 However, there is still no objective consensus regarding the benefits of combined reconstruction. The IKDC and Lysholm functional scores show better results, but these improvements are only statistically relevant in a few studies. 6 9 10 16 19 20 21 23 We observed better IKDC scores after isolated ACL reconstructions in patients with no associated ALL injury, but without statistically significant difference, and significantly better Lysholm scores in patients undergoing isolated reconstruction with no associated ALL injuries.

Since the ALL is a recently characterized structure, the literature clearly shows the need for longer follow-up to determine the presence or absence of long-term benefits from combined ALL and ACL reconstruction with prospective, randomized, controlled clinical studies with a large number of patients. 1 9 14 15 16 20 24 25

The present study has certain important limitations. As a retrospective study, there is a risk of some biases typical of a non-prospective study. A higher number of cases would be the best option to increase the power of the statistical analysis and demonstrate potential differences between the groups. We had 221 patients operated on during the period and included in the study. However, only 103 underwent imaging exams and surgery at the times considered ideal, and we were unable to contact 51 patients. The mean age of the patients in the present study (33.3 years) was above the mean age observed in similar studies (24 years) .11

## Conclusion

Patients undergoing ACL reconstruction with and without an associated ALL injury presented no difference regarding the rates of new injuries or new surgery. Both groups presented similar results regarding satisfaction with the knee, IKDC score, and return to activities. The Lysholm score was better in patients with no associated ALL injury.
