# Supplementary material for: Outcomes of Anterior Cruciate Ligament Reconstruction in Patients with Associated Anterolateral Ligament Injury
Source: Rev Bras Ortop (Sao Paulo). 2024 Jun 22;59(3):e397–402. doi: 10.1055/s-0044-1785516 (PMC11193583; doi:10.1055/s-0044-1785516)
Supplement: Related texts

# Research article: Resultados da reconstrução do ligamento cruzado anterior em pacientes com lesão associada do ligamento anterolateral

**Keywords:** joelho, ligamento anterolateral, ligamentos articulares, ligamento cruzado anterior

## Abstract

**Objetivo** Avaliar se há diferença significativa nos resultados da reconstrução isolada do ligamento cruzado anterior (LCA) em pacientes com e sem lesão associada do ligamento anterolateral (LAL).

**Métodos** Foi realizado um estudo transversal retrospectivo com análise de prontuários e aplicação dos questionários da Escala de Pontuação do Joelho de Lysholm e do Formulário Subjetivo de Joelho do International Knee Documentation Committee (IKDC) a pacientes com reconstrução isolada do LCA.

**Resultados** Os 52 participantes incluídos foram separados em 2 grupos: 19 com lesão associada do LAL e 33 sem lesão associada. Nenhum paciente com lesão associada do LAL sofreu rerruptura do LCA, e 21,1% tiveram lesões em outras estruturas do joelho após a cirurgia. Entre os pacientes sem lesão associada, 6,1% sofreram rerruptura do LCA, e 18,2% tiveram lesões em outras estruturas após a cirurgia ( *p* = 0,544). O retorno às atividades no mesmo nível do que no pré-operatório foi observado em 60% dos pacientes com lesão associada do LAL e em 72% daqueles sem lesão associada ( *p* = 0,309). Na Escala de Pontuação do Joelho de Lysholm, os pacientes com lesão associada do LAL obtiveram média de 81,6 pontos, e os sem lesão associada, média de 90,1 pontos ( *p* = 0,032). No Formulário Subjetivo de Joelho do IKDC, os pacientes com lesão associada do LAL obtiveram média de 70,3 pontos, e os sem lesão associada, média de 76,7 pontos ( *p* = 0,112).

**Conclusão** Não foi observada diferença estatística significativa quanto a lesões do enxerto ou novas lesões de outras estruturas, satisfação com o joelho operado ou pontuação no Formulário Subjetivo de Joelho do IKDC. O retorno às atividades foi semelhante nos grupos com e sem lesão associada do LAL, e os resultados na Escala de Pontuação do Joelho de Lysholm foram melhores, com diferença estatística significativa no grupo sem lesão associada do LAL.

## Introdução

As lesões do ligamento cruzado anterior (LCA) são muito frequentes, e têm sua incidência aumentada na população fisicamente ativa, com consequências importantes na qualidade de vida dos pacientes, pois afetam o índice de atividade, a estabilidade articular, a funcionalidade e o risco de desenvolvimento de osteoartrite. 1 Entretanto, apesar de a reconstrução cirúrgica ser o tratamento de escolha, a taxa de sucesso é influenciada por fatores de risco individuais do próprio paciente ou do enxerto ligamentar propriamente dito. 1 2

Visando minimizar tais riscos e buscar um tratamento mais eficaz, nos últimos anos, diversas pesquisas 3 4 buscaram caracterizar melhor o complexo anterolateral do joelho, e demonstrou-se que o ligamento anterolateral (LAL) exerce importante função estabilizadora para a rotação interna do joelho, assim como o LCA. Nesse contexto, observou-se que lesões associadas do LCA com o LAL apresentavam piores resultados após a reconstrução cirúrgica do LCA isoladamente, e argumenta-se a necessidade de se associar o procedimento extra-articular à reconstrução intra-articular. 5 6

Com base nessas informações, o presente trabalho buscou analisar de forma retrospectiva os resultados da reconstrução isolada do LCA em grupos de pacientes diagnosticados com a lesão do LCA associada ou não à lesão do LAL.

## Materiais e Métodos

O presente estudo foi realizado após aprovação pelo Comitê de Ética em Pesquisa (CAAE- 61209722.0.0000.0020) da instituição mediante consentimento assinado pelos participantes, e está de acordo com a resolução n° 466/2012 do Conselho Nacional de Saúde e a Declaração de Helsinque.

Foram realizadas análise de prontuários e aplicação dos questionários da Escala de Pontuação do Joelho de Lysholm e do Formulário Subjetivo de Joelho do International Knee Documentation Committee (IKDC), nas suas versões traduzidas para a língua portuguesa, 7 8 em todos os pacientes submetidos à reconstrução isolada do LCA pelo mesmo grupo de cirurgia de joelho em 2019.

Foram incluídos pacientes com acompanhamento de pelo menos dois anos, que tinham exames de ressonância magnética realizados na fase aguda da lesão (até três semanas depois da entorse inicial), e operados nos primeiros três meses após a lesão. Foram excluídos pacientes que se recusaram a participar da pesquisa ou que não puderam ser contactados.

Nos prontuários, foi foram coletadas informações acerca do sexo, da data de nascimento, da data da lesão, da data do exame de ressonância pré-operatório, da data da cirurgia e do lado operado. Os pacientes selecionados foram submetidos a uma entrevista e ao preenchimento dos questionários. Os participantes responderam inicialmente se tiveram nova lesão do LCA ou de outra estrutura com indicação de cirurgia no mesmo joelho. Os que não tiveram novas lesões foram questionados sobre o grau de satisfação com a cirurgia (muito satisfeito, satisfeito, pouco satisfeito ou insatisfeito), sobre o retorno às atividades esportivas (melhor do que antes, igual a antes, pior do que antes ou não conseguiram retornar) e foram aplicados os questionários de função da Escala de Lysholm 7 e do IKDC. 8 Os pacientes com novas lesões foram excluídos dessa etapa por não se enquadrarem nos critérios de acompanhamento mínimo de 2 anos no pós-operatório.

## Análise Estatística

A análise estatística das variáveis qualitativas foi feita por meio do teste do qui-quadrado (χ ^2^ ) ou exato de Fischer. Para as variáveis quantitativas, foi aplicado o teste de Shapiro-Wilk para a análise da normalidade, e, posteriormente, o teste de Mann-Whitney para dados não normais e teste *t* para variáveis com distribuição gaussiana. Os resultados foram analisados pelo programa IBM SPSS Statistics for Windows (IBM Corp., Armonk, NY, Estados Unidos), versão 23.0, sendo estabelecido um nível de confiança de 5% para todos os testes aplicados.

## Resultados

A partir da amostra inicial de 221 pacientes submetidos à reconstrução do LCA, foram incluídos 103 participantes que tinham exames de ressonância magnética realizados nas primeiras 3 semanas após a lesão e operados em até 3 meses após a lesão. Acabaram sendo excluídos 1 paciente que se recusou a participar da pesquisa e 50 pacientes com prontuários incompletos e que não conseguiram ser contatados. Os 52 participantes avaliados foram divididos em 2 grupos conforme a presença ou ausência de lesão do LAL no pré-operatório da cirurgia de reconstrução do LCA ( Fig. 1 ).

**Fig. 1:** Fluxograma do estudo.

Em relação à idade, observamos média de 33,3 anos no grupo com lesão associada de LAL e 38 anos no grupo sem lesão associada do LAL, com predominância do sexo masculino (73,7% do grupo com lesão do LAL e 87,9% do grupo sem essa lesão. Com relação às novas lesões, após 2 anos, observamos que nenhum paciente com lesão do LAL teve nova lesão do LCA e que 21,1% dos pacientes haviam lesado outra estrutura do joelho (menisco, cartilagem ou outro ligamento), ao passo que, entre os pacientes sem lesão do LAL, 6,1% tiveram nova lesão do LCA e 18,2%, lesões em outra estrutura ( Tabela 1 ).

**Tabela 1:** Idade, sexo e novas lesões

Variável	Lesão no LAL (n = 19)	Sem lesão no LAL (n = 33)	Valor de *p*
Idade (anos): média ± DP	33,3 ± 8,8	38 ± 10,4	0,243
Sexo masculino: n (%)	14 (73,7)	29 (87,9)	0,260
Nova lesão no joelho: n (%)			
- Sim, nova lesão do LCA - Sim, mas de outra estrutura - Não teve nova lesão	0 (0) 4 (21,1) 15 (78,9)	2 (6,1) 6 (18,2) 25 (75,8)	0,544

Abreviaturas: DP, desvio padrão; LAL, ligamento anterolateral; LCA, ligamento cruzado anterior.

Quanto aos pacientes que não apresentaram nova lesão do joelho, observamos média de idade de 34,4 anos para aqueles com lesão associada do LAL e 39,6 anos para aqueles sem essa lesão, ainda com predomínio do sexo masculino (73,3% do grupo com lesão do LAL e 84% do grupo sem essa lesão). Quanto à satisfação com o joelho operado, em ambos os grupos houve maioria de pacientes muito satisfeitos, sendo 46,7% com lesão do LAL e 52% sem lesão do LAL ( *p* = 0,367).

Sobre o retorno às atividades, 60% com lesão do LAL e 72% sem lesão do LAL ( *p* = 0,309) retornaram ao mesmo nível de antes da operação.

Quanto aos questionários de função, na Escala de Lysholm houve média de 81,6 pontos nos pacientes com lesão do LAL e de 90,1 pontos naqueles sem lesão do LAL ( *p* = 0,032), e no IKDC, 70,3 pontos nos pacientes com lesão do LAL e 76,7 pontos nos sem lesão do LAL ( *p* = 0,112) ( Tabela 2 ).

**Tabela 2:** Satisfação, retorno às atividades esportivas e questionários de função

Variável	Lesão no LAL (n = 15)	Sem lesão no LAL (n = 25)	Valor de *p*
Idade (anos): média ± DP	34,4 ± 9,4	39,6 ± 10,5	0,118
Sexo masculino: n (%)	11 (73,3)	21 (84)	0,444
Satisfação: n (%)			
- Muito satisfeito - Satisfeito - Pouco satisfeito - Insatisfeito	7 (46,7) 5 (33,3) 3 (20) 0 (0)	13 (52) 10 (40) 1 (4) 1 (4)	0,367
Retorno às atividades: n (%)			
- Melhor do que antes - Igual a antes - Pior do que antes - Não conseguiram retornar	2 (13,3) 9 (60) 4 (26,7) 0 (0)	3 (12) 18 (72) 2 (8) 2 (8)	0,309
Escala de Pontuação do Joelho de Lysholm: média ± DP	81,6 ± 18,6	90,1 ± 15,5	0,032
Formulário Subjetivo de Joelho do IKDC: média ± DP	70,3 ± 15,4	76,7 ± 14,3	0,112

Abreviaturas: DP, desvio padrão; IKDC, International Knee Documentation Committee; LAL, ligamento anterolateral.

## Discussão

Ao analisar o índice de nova lesão do enxerto do LCA, no presente estudo não se observaram diferenças significativas ou necessidade de novas cirurgias nos pacientes de ambos os grupos. Contudo, alguns estudos comprovaram que a lesão associada aumenta as taxas de ruptura do enxerto de LCA 6 9 10 e de reoperação, apesar de um grande estudo retrospectivo 11 com reconstrução combinada não mostrar diferença significativa com relação às reconstruções isoladas do LCA. As desvantagens da lesão associada e as vantagens da reconstrução combinada se dariam graças às propriedades anatômicas e biomecânicas do LAL para a estabilização rotatória do joelho junto com o LCA, e já foi comprovado em estudos em cadáveres 12 13 14 que a biomecânica e a cinética do joelho só são restauradas aos níveis anteriores à lesão na presença de reconstruções combinadas de lesões associadas.

Alguns pesquisadores 14 15 16 17 18 consideram como indicação de reconstrução combinada: a presença de um critério maior para risco aumentado de rerruptura do LCA; *pivot* residual positivo no pós-operatório; ou dois critérios menores para risco aumentado de nova lesão. Entretanto, até o momento, não houve padronização das indicações para a reconstrução extra-articular do LAL associada à reconstrução intra-articular do LCA, com diversos autores 3 5 14 15 16 17 18 atentando para a necessidade de estudos mais robustos sobre o tema.

Com relação ao resultado funcional e à satisfação do paciente após a reconstrução, grande parte dos estudos 10 14 15 16 17 18 19 20 21 22 23 mostram melhoras objetivas e/ou subjetivas com a reconstrução combinada. Entretanto, ainda há muitas controvérsias na literatura, e boa parte desses estudos mostram não haver diferença estatisticamente significativa, exceto quanto à presença de hipermobilidade articular associada a importante instabilidade rotacional do joelho, em que a reconstrução combinada demonstrou melhor satisfação dos pacientes. 10 16 17 19 21 Em relação ao retorno às atividades diárias e práticas esportivas, foi demonstrada melhora funcional com a reconstrução combinada, principalmente em populações com hipermobilidade no joelho; 15 16 17 no entanto, ainda não há um consenso objetivo quanto aos benefícios da reconstrução combinada, e os escores funcionais de IKDC e de Lysholm apresentam resultados funcionalmente melhores, mas que somente são considerados estatisticamente relevantes em alguns estudos. 6 9 10 16 19 20 21 23 Neste estudo, foram observados melhores resultados no questionário do IKDC para reconstruções isoladas do LCA em pacientes sem lesão associada do LAL, mas sem diferença estatisticamente significativa, e melhores resultados no questionário de Lysholm, sendo este estatisticamente significativo a favor da reconstrução isolada em pacientes sem lesões associadas do LAL.

Por se tratar de uma estrutura caracterizada recentemente, a literatura mostra claramente a necessidade de seguimento mais prolongado para a averiguação da presença ou não de benefícios no longo prazo da reconstrução combinada dos ligamentos analisados, e com estudos clínicos prospectivos randomizados e controlados com grande número de pacientes. 1 9 14 15 16 20 24 25

Este estudo apresenta algumas limitações importantes. Por se tratar de um estudo retrospectivo, há risco de alguns vieses típicos de um estudo não prospectivo, e um maior número de casos seria o mais indicado para aumentar o poder da análise estatística e demonstrar possíveis diferenças entre os grupos. Tivemos 221 pacientes operados no período determinado para a inclusão no estudo, mas só 103 fizeram exames de imagem e foram operados nos tempos considerados ideais e, ainda assim, 51 pacientes não puderam ser contactados. A média de idade dos pacientes deste estudo (33,3 anos) ficou acima da média dos estudos sobre o mesmo tema (24 anos). 11

## Conclusão

Nos pacientes submetidos à reconstrução do LCA com e sem a lesão associada do LAL, não foi observada diferença com relação aos índices de nova lesão ou nova cirurgia. Quanto à satisfação com o joelho, o questionário do IKDC e o retorno às atividades, ambos os grupos demonstraram resultados semelhantes. A pontuação na Escala de Lysholm foi melhor entre os pacientes sem lesão associada do LAL.
